# Evidence in the learning organization

**DOI:** 10.1186/1478-4505-7-4

**Published:** 2009-03-26

**Authors:** Gerald E Crites, Megan C McNamara, Elie A Akl, W Scott Richardson, Craig A Umscheid, James Nishikawa

**Affiliations:** 1 Wright State University Boonshoft School of Medicine, One Elizabeth Place, Suite 500, Dayton, OH, USA; 2 School of Medicine, Case Western Reserve University, Louis Stokes Cleveland VA Medical Center, 10701 East Boulevard, Cleveland, USA; 3 School of Medicine, State University of New York at Buffalo, Erie County Medical Center, CC142, Buffalo, NY, USA; 4 Wright State University Boonshoft School of Medicine, Miami Valley Hospital, Weber CHE Building, 2nd Floor, 128 E. Apple St, Dayton, OH, USA; 5 School of Medicine, University of Pennsylvania, 1 Founders Pavilion, 3400 Spruce Street, Philadelphia, PA, USA; 6 Faculty of Medicine, University of Ottawa, 1053 Carling Ave., Room 412 Parkdale Building, Ottawa, ON, Canada

## Abstract

**Background:**

Organizational leaders in business and medicine have been experiencing a similar dilemma: how to ensure that their organizational members are adopting work innovations in a timely fashion. Organizational leaders in healthcare have attempted to resolve this dilemma by offering specific solutions, such as evidence-based medicine (EBM), but organizations are still not systematically adopting evidence-based practice innovations as rapidly as expected by policy-makers (the knowing-doing gap problem). Some business leaders have adopted a systems-based perspective, called the learning organization (LO), to address a similar dilemma. Three years ago, the Society of General Internal Medicine's Evidence-based Medicine Task Force began an inquiry to integrate the EBM and LO concepts into one model to address the knowing-doing gap problem.

**Methods:**

During the model development process, the authors searched several databases for relevant LO frameworks and their related concepts by using a broad search strategy. To identify the key LO frameworks and consolidate them into one model, the authors used consensus-based decision-making and a narrative thematic synthesis guided by several qualitative criteria. The authors subjected the model to external, independent review and improved upon its design with this feedback.

**Results:**

The authors found seven LO frameworks particularly relevant to evidence-based practice innovations in organizations. The authors describe their interpretations of these frameworks for healthcare organizations, the process they used to integrate the LO frameworks with EBM principles, and the resulting Evidence in the Learning Organization (ELO) model. They also provide a health organization scenario to illustrate ELO concepts in application.

**Conclusion:**

The authors intend, by sharing the LO frameworks and the ELO model, to help organizations identify their capacities to learn and share knowledge about evidence-based practice innovations. The ELO model will need further validation and improvement through its use in organizational settings and applied health services research.

## An organizational scenario

You are the new Chief of Quality in a university health center and are studying the inadequate utilization of an evidence-based guideline designed to reduce the number of missed diagnoses and improve the early treatment of sepsis. Your predecessor implemented the guideline with standard implementation strategies, including training providers using the guideline's results, using timely chart audits with individual/unit reports, periodic detailing by an infection expert, identifying and engaging local opinion leaders and integrating reminders into the electronic patient record. These strategies improved the sepsis outcomes, but there is an obvious need to improve early diagnoses and antibiotic utilization. From the audit reports, you note that most units are underperforming on the practice standards. You are mystified how further to improve this situation.

## Background

Research evidence about decision errors of omission, from diagnostic errors to the underutilization of medications, suggests that these errors may be due, in part, to unnecessary delays in knowledge translation and result in needless human suffering [1,2]. Some people have labeled this continuing phenomenon, exemplified in the opening scenario, as the knowing-doing gap problem [3]. Educational solutions, such as Evidence-based Medicine (EBM) and Total Quality Improvement, and organizational change solutions, such as implementation science, attempt to address the knowing-doing gap by improving evidence availability, promoting evidence-based practice skills, and influencing evidence-based practice behaviors within and across healthcare organizations [4,5]. Despite the wide availability of these state-of-the-art solutions, many organizations are still experiencing a knowing-doing gap problem [6].

To address a similar dilemma, successful business organizations respond to market changes by efficiently implementing practice innovations that incorporate market-generated information and make organizational changes to enhance utilization of this knowledge; such organizations are called "Learning Organizations" (LO) [7]. In the business and academic worlds, the LO concept is often extended with three additional organizational principles: organizational learning, organizational knowledge, and knowledge management [8]. Organizational learning is the process of transforming external market information into practical, contextual knowledge that informs practices across the organization [8]. Organizational knowledge is the product of the learning process and includes internal (tacit knowledge, or "know how" knowledge, held only in minds of organizational members) and external (explicit knowledge, such as best practice recommendations) forms [8]. Knowledge management is the control of organizational structures and processes, via communication networks and information systems, to facilitate knowledge sharing across the organization [8]. A successful LO is one that utilizes organizational learning and knowledge management principles while monitoring and managing human systems to facilitate the capture and use of new organizational knowledge.

Because of the similarity of the knowing-doing gap problems in medicine and business, some of the LO solutions learned in business organizations might be applied to the healthcare organizational setting. Indeed, some researchers admit that there are gaps in their knowledge about how healthcare decision-makers use knowledge contextually and adapt their decisions to new information [9-11], and LO frameworks may help to elucidate these processes. If relevant LO frameworks could be consolidated into one model for application in healthcare settings, then implementation experts and healthcare leaders would have a cohesive, theoretical foundation to understand some of the reasons why organizations fail to adopt practice innovations [5,12-18]. Finally, implementation experts who use multifaceted implementation strategies tailored to different organizational contexts may need flexible tools to diagnose implementation targets across these different contexts [16,18,19].

Over three years, a multidisciplinary team of scholars completed a review of LO frameworks, interpreted them for healthcare organizational contexts, and consolidated them into a cohesive model. We describe the LO frameworks and the consolidated model, called Evidence in the Learning Organization (ELO), to provide healthcare leaders, managers, and their employees a practical framework for understanding how their organizations learn and build contextually-based knowledge gained through evidence-based decision making. This model should help organizational leaders and implementation experts identify their organizational flaws with learning and sharing this knowledge and, therefore, identify targets for interventions.

## Methods

### Literature Review

The ELO team consisted of six members of the Society of General Internal Medicine's EBM Task Force who have experience in EBM, medical education, healthcare management, public health, clinical research and quality improvement.

Our first task was to identify the scope of the LO and related literature within and outside of medical databases; thus, our aim was to get a large sample of the concepts by using a broad search strategy. We searched several databases using each database's unique thesaurus and field codes. We identified the following database/strategy combinations most useful:

- In PubMed: "organizational learning [EXPLODE]," "organizational culture [EXPLODE]," "organizational innovation [EXPLODE]," "organizational culture [MeSH] AND evidence-based medicine [MeSH]," "evidence-based medicine [MeSH] AND organizational innovation [MeSH]," "informatics [MESH] AND organizational Innovation [MeSH]", and "models, organization [MeSH] AND evidence-based medicine [MeSH]."

- In Business Source Primer: "organizational learning [EXPLODE]," "organizational learning [TX] AND Knowledge Management [TX]," "organizational learning [TX] AND organizational change [TX]," "organizational learning [TX] AND culture [TX]," and "organizational learning [TX] AND organizational behavior [TX] AND knowledge management [TX]."

- In Sociological Abstracts: "organizational learning [KEY WORD]," "organizational culture AND organizational learning [SUT]," and "organizational culture [SUT] AND organizational development [SUT]."

- In SocINDEX: "organizational learning [EXPLODE]," "organizational learning [SU] AND corporate culture [SU]," and "organizational change [SU] AND corporate culture [SU]."

We used several of the search strategies described above with the ERIC database but were unable to identify any useful references.

In addition to the database searches, we searched the indices for one journal, *The Learning Organization*, back to its inception (1994). We also identified key texts from both academia and consultative business practitioners by cross-referencing these texts from resources identified in the search. We engaged several experts in various fields (health systems research, leadership, and quality improvement) to identify key works in organization leadership and management. We continued to review the literature until we consistently failed to identify new theories, frameworks, models, or concepts related to the LO principles (information saturation).

### Framework selection

Because we were searching for the best theoretical LO frameworks and their constituent concepts to support the EBM and LO integration, we used consensus-based decision-making to select the relevant frameworks. We identified several qualitative criteria to assist our decisions: how seminal a framework was in establishing a new line of inquiry; how frequently a framework was cross-referenced in the literature; how representative a framework was for healthcare organizational applications; how well empiric research supported a framework; how well a framework fit with other frameworks, and how well a framework appealed to team members' intuition and experience. We reviewed all frameworks and their concepts independently and later, during a retreat, built consensus around our final choices. During the retreat, each ELO team member described his/her reasoning for choosing specific frameworks and concepts using the criteria. The other team members would subject these arguments to critical discourse and offer opportunities for rebuttal. The discourse continued until all team members could agree on which frameworks and constituent concepts to include in the review.

### Model consolidation process

To consolidate the selected frameworks into one model, we used a narrative thematic synthesis, rather than a quantitative systematic review. For this consolidation phase, we identified a series of candidate ELO models and subjected them to the same consensus process described above. After reaching consensus on the best candidate model, we subjected it to a second review process by engaging leaders in medical management, quality control, medical sociology and health systems scholarship to review the model for clarity, representativeness, and inclusiveness. We updated the model with the feedback from these experts.

## Results

### Selected LO frameworks

From our literature review, we developed a glossary to guide our discourse (Appendix 1) and identified seven key LO frameworks that best informed healthcare contexts (Table 1). We excluded several frameworks and models because we could not build consensus around their inclusion [8,20-41]. There were several reasons for this lack of consensus: we had difficulty in interpreting some models into healthcare organizational contexts or they did not fit well with earlier selections [8,21,22,26,28,30,33,37,39,41]; some models were more focused extensions of seminal works [8,20,25,29,38,40], and; some models were cited by infrequently by other references and had little applied research to support them [22-24,27,31,35,36].

**Table 1:** Seven Informative Learning Organization Frameworks and Their Key Concepts

	**Learning Process:**	**Who creates the knowledge?**	**Knowledge is validated through:**	**Organizational model:**	**How evidence fits into each framework?**
**Organizational Learning **[42,44]	Loop Learning and the "4i"	Individual to Organization	Applying a Work Strategy or Process	Individuals as Agents for Organizational Inquiry	Evidence as External Strategy To Consider

**Decision-Execution Cycles **[45]	Testing Knowledge Gaps During Work	Individual to Organization	Knowledge Claims Tested Through Decisions	Knowledge Formed in Minds & Information Systems	External Knowledge Claim To Be Tested

**Organizational Knowledge Creation **[46]	Team Discourse: Making Tacit Knowledge Explicit	Individual and Team; then to Organization	Negotiated Understanding of Decisions in Action	Social Network of Relating Teams	External Knowledge To Be Justified by Working Teams

**Organizational Culture **[52-56]	Cultural Change Processes & Assimilation	Organization & Organizational Subcultures	Negotiated, Shared Beliefs of Organizations & Subcultures	Vocational Society and Subcultures	Evidence as Data to Confirm/Disconfirm Beliefs

**Complex Adaptive Systems **[57-61]	Decentralized Decision-making with Rules & Policies	Not Stated or Implied	Not Stated or Implied	Complex System (of Providers & Patients)	Evidence as New Policy or Practice

**Diffusion & Dissemination of Innovation **[37,62]	Diffusion or Active Dissemination Strategies	Not Stated or Implied	Adoption of Practice Innovations	System of Adopters with Variable Readiness for Change	Innovative Concept or Practice to be Adopted

**Total Quality Management (TQM) **[24,33]	Redesign of Individual Work Processes	Leaders & Practitioners	Practice Standard or Benchmark Attainment	Mechanical System (of Linear Processes)	External Benchmark or Standard Practice

#### Two organizational learning frameworks

The first three frameworks in Table 1 were useful in elucidating the organizational learning and organizational knowledge creation processes. Argyris and Schön originally described the organizational learning (OL) concept and created the *loop learning *framework to demonstrate how organizational members, acting as agents for organizational inquiry, assist organizational learning with three cognitive tasks: 1) learning if work processes are adequate to implement current strategies (single-loop, or "adaptive" learning), 2) learning if the current organizational assumptions about strategy effectiveness are valid (double-loop, or "generative" learning), and 3) studying the effectiveness of organizational learning structures and processes (deutero-loop learning, also described as triple-loop or meta-learning) [42,43].

Although Argyris and Schön's seminal loop learning framework provides a general understanding of organizational learning, it needs clarification on how it flows across different organizational levels. Crossan's *"4i" *framework (intuiting, interpreting, integrating, and institutionalizing) extends the description of loop learning processes across three organizational levels: members sense and capitalize upon organizational learning opportunities (intuiting to interpreting); teams, using the results of these learning processes, discuss and create a collective understanding for needed changes (interpreting to integrating); and organizations capture and share this new knowledge to benefit all its members (integrating to institutionalizing) [44]. Once the 4i cycle is complete, organizational knowledge is "renewed" and updated [44]. Thus, individuals, acting as agents of organizational inquiry, recognize and capitalize upon loop learning opportunities, teams help interpret the results of loop learning and integrate it with previous knowledge, and organizations monitor activities to capture and share this knowledge though detuero-loop learning management.

We can illustrate the loop-learning and 4i frameworks by describing a hypothetical initiative designed to improve Prostate Specific Antigen (PSA) testing decisions for prostate cancer screening. An organization can discover, through routine audit mechanisms, that providers' documentation show unacceptably low frequencies of orders for PSA tests. After implementing changes to improve work processes, managers determine, through further audit and reporting cycles, if the changes result in improved compliance with the practice standard. When organizations delegate to teams the responsibility for deliberating (interpreting) upon the team members' learning opportunities (intuiting) and forming recommendations about the needed process changes (integrating), the organization is adept at practicing single-loop learning at the team level. Single-loop learning provides organizations opportunities to learn more about the practical limitations of specific policies and how working teams can help provide feedback about these limitations.

Despite adapting adequate processes of care, teams may find through the same intuiting/interpreting/integrating processes that their patients are declining PSA tests at a high frequency because their patients desire more knowledge, discussion time, and decision options for all the complicated prostate screening issues, including their costs and consequences. In this example of double-loop learning, the organization should rethink its priorities and adapt its strategies accordingly, such as offering providers more time to discuss PSA decisions with patients, providing patients more education on screening decisions or resetting their benchmark targets to reflect more realistically on their patients' preferences. Finally, organizations may discover that they have inadequate structures or processes to facilitate sharing this new knowledge; for example, organizations may discover that no one is tasked to filter and reformulate knowledge gained from single- and double-loop learning at the management or committee level (deutero-loop learning). To be successful at single-, double- and deutero-loop learning, organizations must have adequate resources and processes to capture loop learning and flexibility to adapt policies when the assumptions underlying these policies are proven incorrect.

#### The Decision-Execution Cycle Framework

Firestone and McElroy's *Decision-Execution Cycles *framework describes more specifically how organizational members use the intuiting process as an opportunity for loop learning by testing recommendations from new (e.g., a new evidence-based recommendation) or old practice policies, called knowledge claims, with patient care decisions. In this process, members compare the expected outcomes of their decisions to the actual ones (in the form of qualitative experience or quantitative feedback) and form epistemic gaps, or "a-ha" moments, as learning moments about specific knowledge claims [45]. The knowledge gained through epistemic learning can be added to the organizational knowledge base, which is held collectively in members' minds and information systems [45].

In their analysis of the Partners HealthCare case study, Firestone and McElroy described how physicians in Partners HealthCare, when provided with electronic prescribing alerts integrated with the electronic patient record, improved compliance with prescribing standards by challenging the physicians to rethink the assumptions of their decisions [45]. Because the system also required physicians who overrode the prescribing alerts to provide reasons for the override actions (as they did 28% of the time), physicians could provide feedback to the information system and improve the collective understanding about specific prescribing decisions [45]. The hospital committee assigned to manage the alert system would reformulate this feedback into more sophisticated reminders and add them to the information system, thus renewing the existing organizational knowledge base [45]. The DEC framework implies that organizations that provide decision-makers with a flexible, interactive and adaptive knowledge system with viable decision options (and their assumptions) may help better support loop learning and decision-making at the point of service.

#### Organizational knowledge creation framework

Sometimes external knowledge claims need to be translated into working knowledge through discourse prior to their use. Nonaka and Takeuchi's *organizational knowledge creation *framework helps clarify the team learning (interpreting/integrating) process by describing how "know-how" knowledge, called tacit knowledge, is continually built, used, and reformulated [46]. Within this framework, any practice policies coming from outside the team structure (e.g., an evidence-based policy) must be validated through informal team discourse and modified for practical applications [46]. Team members then use this tacit knowledge with work applications and form, through further team discourse, a clearer and a more explicit description of its practical applications and limitations [46]. Teams often share these reformulated recommendations across an informal network of working teams, which provides another avenue for validating the recommendations [46]. Professional networks that help validate and clarify policy and practice recommendations for practical application are called "communities-of-practice." [47].

An example of the organizational knowledge creation framework comes from Gabay and Lemay's ethnographic "mindlines" study. These investigators studied how primary care doctors and nurses used external evidence-based guidelines to create "mindlines," which were internally-based, tacit guidelines that the providers socially-constructed through an iterative process [48]. For example, one physician who wanted an updated protocol for heart failure management synthesized the protocol from two published guidelines, recommendations from local cardiologists, and her local practice patterns [48]. She then presented this modified protocol to other members of her practice, who debated its utility based upon its flexibility to fit into the team's practice patterns, the experiences of other local care teams, the feasibility of practice changes required for its success, and its likely impact on practice finances and healthcare quality [48]. Several local events could trigger further team discourse and modify the mindlines, including critical incidents, new practice barriers, unique anecdotes, patient opinions, and audit reports [48]. These providers' believed the mindlines constructed this way to be highly authoritative, even more so than the broader organizational policies [48]. Organizational leaders who understand how their patient care teams socially-construct their own care guidelines may be in a better position to understand cross-unit variability in decision-making and influence these local practice constructs.

#### Organizational culture framework

The last four frameworks in Table 1 provide systems-based perspectives for understanding potential facilitators or inhibitors to organizational learning and knowledge management. Schein notes that organizational members work under shared assumptions expressed as the beliefs and norms of the *organizational culture*, and these cultural influences can impact their acceptance or resistance to organizational learning [49-52]. For example, organizations that have members who collaboratively make decisions in teams, show a willingness to experiment with innovation, question policy and practice assumptions, share cohesive team values, and are committed to improvement have a higher frequency of research use, adapt faster to innovations, and possibly improve healthcare quality [9,19,53-55]. Hierarchal organizations that display cultures of centralized decision-making and directive leadership seem to inhibit organizational learning and new knowledge formation [30,37]. If the prevailing organizational cultural climate is resistant to learning, then only coordinated efforts by leadership, using strategies such as organizational restructuring, emphasizing performance discordance, championing change, and using incentives that promote adoptive practices, can induce cultural change [52].

Beyond broad organizational cultural beliefs that may inhibit decentralized learning, specific subculture conflicts may exist in organizations and cause variable adoption of evidence-based practices. For example, the subcultural values of autonomy held by physicians or pragmaticism held by nurses may be sources of resistance to change [30,56]. Organizational leaders who fail to negotiate these variable belief systems within organizations may risk failure with implementation initiatives.

#### Complex Adaptive Systems Framework

The *Complex Adaptive Systems *(CAS) framework views organizations as a group of semi-autonomous decision-makers who try to reconcile best practice recommendations (minimum specifications) and their policies (attractors) with the complex, local influences on healthcare delivery (additional attractors), including the contextual barriers to decision-making and the beliefs of co-workers, management and patients [57-59]. As they negotiate how to work together in teams to face these continually evolving challenges, decision-makers develop locally coordinated practice patterns (self-organization) around these attractors and minimum specifications [60]. The implication of the CAS framework is that these local influences on decisions, some of which we described earlier, are best identified and managed locally by teams and their managers and evidence can serve as a behavioral influence if these decision-makers perceive it as a strong attractor (policy) or minimum specification (best practice recommendation).

In their description of an a new initiative at a United Kingdom Primary Care Trust, Rowe and Hogarth illustrated their attempt to influence more responsive public health decision-making using CAS as a guiding framework [61]. The Trust managers historically used multiple, centrally-generated policies, procedures, and outcome measures as their primary decision tools and procedures [61]. In order to become more responsible to the diverse communities it served, the Trust leadership recognized the need to decentralize decision processes and delegate the decisions about health priorities, initiatives and processes to local practitioners [61]. Each primary care site personnel generated, through team reflection and debate, a common understanding of the needed health priorities for their immediate communities using variety of attractors: valuing generative relationships with the community and other practice sites, shared accountability for outcomes in their catchment area, multidisciplinary input, experimentation and innovation, beliefs of local providers, and cooperation between teams and management [61]. The public heath personnel also simplified principles from numerous health policies into a few minimum specifications to guide their practices [61]. The results of this initiative included a wide variety of new health service lines and processes across practice sites to address immediate community needs, from targeting the needs of specific patients, such as an outreach clinic for asylum seekers, to community-focused programs, such as an exercise class for a particular ethnic group (self-organization) [61].

#### Diffusion and dissemination of innovation frameworks

*Diffusion and dissemination of innovation *are two closely related perspectives that help us understand how evidence-based practice innovations are adopted passively and variably by members and teams (diffusion) or can be accelerated thorough intentional organizational strategies (dissemination) [37,38,62,63]. These perspectives have identified several characteristics about individuals, teams, and organizations which can predict their readiness for change. In their study of seven patient care units from four Canadian hospitals, Estabrooks et al found that the units with higher clinical research utilization clustered around several cognitive domains: positive attitudes towards research use, values of work creativity and efficiency, co-worker support, critical thinking, a willingness to question work behaviors, expressing the importance of continuing education (i.e., learning), perceived authority to implement practice changes, perceived organizational (i.e., leadership) support and the ability to suspend prior beliefs [9].

A systematic review on diffusion and dissemination research from several research fields (business, medicine, social ecology, etc.) has identified additional characteristics about readiness for innovation adoption [37]. An individual's readiness for innovation is reflected by his or her intellectual skills and traits that, in the context of this narrative, we interpret as technical skills (e.g., computer skills) and cognitive skills/traits (e.g., EBM skills, attitudes towards using evidence and intrinsic learning motivation) [37]. Organizational antecedents for successful innovation dissemination include: decentralized decision-making processes; availability of slack resources (including time); professional networks of semiautonomous, specialized teams; strong leadership for change; a risk taking culture (experimentation); a high degree of innovation/cultural match; and the ability to form, capture and share socially-constructed knowledge [37].

These multiple lines of inquiry have been fairly consistent in identifying the types of cognitive, attitudinal, cultural, relational and resource characteristics that promote readiness for innovation adoption within organizations, and many of these characteristics are reflected in earlier frameworks. One barrier to implementing this research is the lack of knowledge about how these characteristics fit together to predict adaptability by decision-makers and teams.

#### Total Quality Management Framework

The *Total Quality Management *(TQM) framework views evidence-based decisions as mostly linear logic models intended to improve health outcomes by re-designing care processes and measuring success through achievement of practice standards and benchmark targets [64]. The TQM approach has been valuable in helping practitioners identify local process changes and tools to improve decision-making [24]; thus, TQM fits nicely into Execution portion of DEC cycle. In the Partners HealthCare case study, the reminder system was a valuable decision process to assist physicians to question their assumptions and expected outcomes. The TQM approach can also be useful for supporting single-loop learning; in the PSA example, when patient care teams deliberated and created better decisions around processes and outcomes, they became adept single-loop learning. However, TQM, in its theoretical formulation, is insufficient to support other forms of loop learning [24,33,64,65]. In the PSA example, only the practice teams could identify patient considerations that required changes in expectations for the standard practices; maintaining executive control of these types of decisions risked losing these learning opportunities. In these instances, organizational leaders typically use knowledge from their own experience to form beliefs about necessary healthcare strategies, priorities, and initiatives, and these beliefs serve the basis for best policy or practice recommendations. This is not to say that quality managers cannot organize successful double-loop learning processes; it is likely that, whenever the limitations of theory meet practicality, these successful managers draw upon other perspectives than TQM to fill the performance gaps [24,33,47].

### Model description

We consolidated these frameworks into the ELO model to assist organizational leaders and scholars with the task of diagnosing organizational learning and knowledge sharing flaws. The ELO model is constituted of four themes and their subthemes that represent the processes required for organizations to learn and share new knowledge more effectively. These processes are not necessarily sequential but can occur simultaneously and interactively:

*1) Inquiring: *Are members are ready to inquire on behalf of teams/organizations to facilitate the loop learning processes?

a. Acquiring: Do they possess technical skills related to locating resources and communicating feedback about this inquiry (e.g., IT training)?

b. Informing: Do they possess the cognitive skills (i.e., EBM skills) that support evidence-based decisions?

c. Transforming: Do they possess cognitive traits that facilitate behaviors for inquiry (e.g., internal learning motivation)?

*2) Deciding: *Are members and teams utilizing effective decision processes to integrate evidence into healthcare decisions?

a. Deliberating: Are they comparing and analyzing new working goals/strategies and structures/processes that will lead to better decisions (e.g., weighing alternative work procedures)?

b. Decision-taking: Are they using appropriate decision methods/tools to support better decision-making making (e.g., computer assisted decision tools)?

c. Evaluating: Are they using adequate analytical methods (qualitative or quantitative) to measure outcomes of evidence-based decisions (e.g., adequate audit and feedback)?

3) *Relating: *Are members, teams, and organizations facilitating evidence-based practices through effective organizational communication and relationships?

a. Sharing: Do the organizational communication structures and processes facilitate sharing knowledge (e.g., adequate information networks)?

b. Cooperating: Are teams available and functioning to facilitate efficient knowledge generation and evaluation (e.g., team composition and roles)?

c. Advocating: Is there adequate and sufficient leadership with effective motivational strategies to induce organizational cultural change towards learning (e.g., incentives, championing, leadership style, etc.)?

*4) Interpreting: *Are members and teams sensing the need for evidence-based practice innovations and explicitly describing their tacit knowledge?

a. Judging: Are they properly evaluating judgments about the outcomes of decisions and needed practice changes (i.e., testing for epistemic gaps)?

b. Knowing: Are they building new models of shared understanding based upon the results of evidence-based decision-making (i.e., interpreting/integrating with communities of practice)?

c. Formulating: Are they codifying this new knowledge (e.g., team-tested practice recommendations) for organizational consumption?

### ELO model application

**Figure 1:**
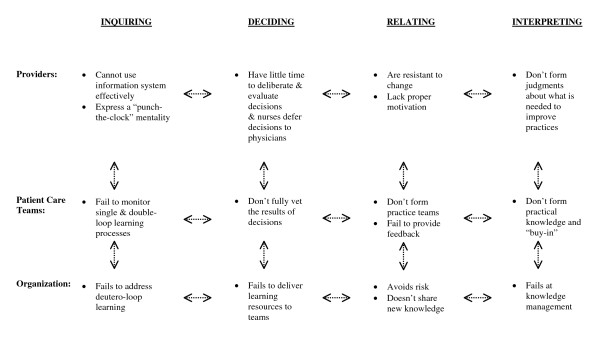
**Application of the Evidence in the Learning Organization Model to a Healthcare Scenario**.

To illustrate the application of this model, we return to our opening scenario to diagnose what went wrong (Figure 1):

With the ELO model in hand, you, the Quality Chief, do further investigation to discover why the guideline initiative failed. Over three days, you talk with providers from patient care units, observe their practice patterns, and review their charts. Additionally, you interview several individuals from leadership, management and hospital committees. After completing your investigation, you find several flaws that may be amenable to specific interventions:

1) *Inquiring difficulty: *Some physicians have yet to learn the reminder system and seem unmotivated to learn it or information shared through the other implementation strategies; thus they lose opportunities to adapt their practices, provide feedback about practical limitations of the initiative (e.g., physician availability to confirm quickly the sepsis source) or to recognize when certain reminders are clinically inappropriate (flaws of acquiring and transforming). Additionally, nurses from several nursing units possess a "punch-the-clock" norm, through which team members are discouraged by influential senior staff members to challenge team leadership with ideas about improving patient care (flaw of transforming).

Because of lost opportunities to improve decision-making processes (single-loop) and to address the quality of the clinical reminders and their limitations (double-loop), teams do not have the capacity to take advantage of these learning opportunities. Also, the committee that oversees the reminder system fails to integrate into it the practice innovations identified by teams that enhance early diagnosis and to adjust the benchmarks with feedback about inappropriate reminders (flaw of deutero-loop learning). The latter flaw has led, in some cases, to an overestimation of the number of missed diagnoses.

2) *Deciding difficulties*: Because of time constraints, providers have little time to review the results of audit reports (flaws of deliberating). Also, the hospital's nursing subculture admires some physicians more than others, and many nurses defer decisions to these physicians (flaws of decision-taking). Unfortunately, these physicians are largely responsible for delayed empiric antibiotic courses and have been identified as local opinion leaders by a recent survey.

Because the diagnosis and treatment decisions are mostly made by under-informed individuals (physicians), teams are not fully vetting decision evaluations and needed changes (flaws of evaluating). Organizational leaders and management committees, unaware of these flaws, make uninformed strategic resource decisions (e.g., time management and team information needs).

3) *Relating Difficulties: *Because of the physicians' inattentiveness to reminders, nurses do not provide feedback to the system about the practical limitations of certain recommendations and when reminders are inappropriate (flaw of sharing). Also, instead of using performance-based incentives, middle managers have used other more controversial tactics to motivate non-compliant physicians, including threats to modify hospital privileges (flaws of advocating). These tactics conflict directly with the autonomy beliefs held by physicians, and this conflict has led more resistance to change.

Because the patient care teams work under these contextual constraints, there are no opportunities for meaningful team discussions about the potential advantages of the guideline, how to modify local practices to achieve its general goals, and address its contextual limitations (flaws of cooperating). Additionally, the hierarchal organizational culture inhibits senior managers delegating process decisions to teams and creating incentives at the team level (additional flaws of advocating). Despite its potential as a flexible knowledge repository, the current IT system is currently managed only for information exchange and, therefore, the organization cannot share new knowledge (additional flaw of sharing).

4) *Interpreting Difficulties: *Because of impairment of individual learning and decision-making, providers are unable to generate accurate judgments about what is happening with their practices and what, if any, changes should occur. Teams cannot form and validate explicit practice recommendations based on these experiences and thus they fail to "buy-into" the initiative. Because of its resource structure and leadership, the organization is failing at knowledge management. The consequences of all these flaws, when magnified at three organizational levels, result in the failure to build and share new organizational knowledge.

During your investigation, you do identify a unit that seems to more successful with this initiative than others. This team consists of technically (IT, communication and EBM) sound providers and local opinion leaders who encourage the norms of inquiry, experimentation, open dialogue, and critical debate. The collective beliefs that guide these norms are shared by most team providers (including physicians) and seem to have helped the team members in adapting to audit reports, detailing, and reminders when these measures suggest improvements are needed. This team is also somewhat successful at identifying inappropriate reminders and adapting their processes to address the initiative's local limitations (such as distance and communication barriers between the unit and the central pharmacy), but they have not experienced a push-back from organizational leadership for variations with standard practices on these issues. The managers who oversee this unit have been adept at shielding the team from this push-back, using incentives to induce practice changes (e.g., paid leave), and using a collaborative leadership style by delegating decisions about process changes to their teams (at their own peril). These managers also help share knowledge gained through team deliberations with other shifts, use creative management techniques to improve time efficiencies to support team discourse, and use their available budgets to improve the technologies (IT system) that support learning and sharing of knowledge across the unit.

We need to make three points about our description of this scenario as a representative ELO model application. First, we described the scenario narrative in a linear manner, but the processes that compose the four themes interact and influence each other in a complex and iterative way (represented by the bi-directional arrows in Figure 1). Second, the scenario reflects barriers, such as cultural resistance and lack of readiness for change, that create flaws across all themes. Third, because the model's utility is broadly applied to identify learning flaws, additional contextual details and other limiting factors may need to be identified prior to forming intervention tactics and strategies.

Because the ELO model is built for in-depth analysis, organizational leaders may need a more concise method to screen for organizational learning, so we have developed eight questions about organizational learning competencies that can be applied to teams or a broader organization. We have used the exemplary unit described in the scenario to demonstrate how to use these screening questions (Appendix 2). In this case, the answers for all eight questions were "yes"; therefore the Quality Chief could quickly bypass this unit to focus her efforts on more flawed units. If any of the answers to the questions are "no," then the unit or organization may have flaws in its learning competencies that could be, in part, contributing to the failures. In this case, leaders would need a broader framework, such as the ELO model, to perform a more thorough analysis.

## Discussion and conclusion

In summary, we described the LO frameworks that we believe relevant for understanding how healthcare organizations learn, create, and share knowledge about evidence-based practices and the system issues that facilitate or inhibit these learning processes. We also describe the ELO model as a cohesive framework to help organizations and implementation experts diagnose flaws in their organizational learning and knowledge management.

From the implementation perspective, the ELO model's weakness may be that it only helps identify targets (monitoring the system) for organizational change, not in its ability to identify specific implementation strategies or tactics to reach this end (managing the system). Fortunately, the research about useful organizational interventions that help facilitate evidence uptake is growing, and we refer readers to a sample of this literature [12,37,66-69]. Ultimately, we see the ELO and implementation science as complementary and convergent perspectives that better inform each other. We also believe that the ELO model could help identify areas for fruitful research where current implementation evidence is scant (as in the case for organizational culture change strategies).

The strengths of our model is its inclusiveness of LO topics, thoughtful consolidation, theoretical grounding, and flexibility to be applied in naturalistic organizational contexts. In the future, we aim to use this model as a framework for research on ideal ELO organizational structures and processes. For example, we intend to use this ELO model to organize and guide the inquiry into the characteristics that are expressed by excellent healthcare organizations and compare them to less successful organizations. This inquiry should both uncover these organizational features and guide refinements and improvements in the ELO model itself. The model also needs validated through its application in naturalistic organizational settings and feedback about its practical application should lead to further refinements.

## Competing interests

Some expenses related to this study were supported from an internal, unrestricted educational fund from the Society of General Internal Medicine.

## Authors' contributions

GEC, MCM, EAA, and WSR contributed to the literature review, identification of frameworks, model synthesis and manuscript authorship. CAU and JN contributed to the model synthesis and manuscript authorship.

## Appendix 1 – glossary of relevant terms

1. **The 4i Model (intuiting, interpreting, integrating, and institutionalizing) **[44]: The 4i demonstrates loop learning by showing how: 1) organizational members reflect on the results of patient care decisions and share this experiences with other team members (intuiting/interpreting), 3) teams form a collective understanding of required practice changes (interpreting/integrating), and 4) organizational leaders disseminate this new knowledge to the organization (integrating/institutionalizing). Members begin this process, called organizational knowledge renewal, by reflecting on failures or successes of old work routines or by using practice innovations championed by leadership (such as in an evidence-based practice guideline).

2. **Communities of Practice **[47]: Sponsored or spontaneously formed teams of practitioners who deliberate on practice issues and form a shared understanding of needed practice changes. These teams can be formed within clinical units, between units, or between organizations.

3. **Complex Adaptive Systems (CAS) **[57]: CAS theory attempts to identify general patterns of in behaviors in complex systems, including human systems. Complex human systems are composed of autonomous/semi-autonomous agents whose behavior patterns are non-linear (i.e., dynamic), interactive, and difficult to predict. Organizational behaviors may move towards chaos, so organizational members tend to react by self-organizing their behaviors around attractors (self or external imposed guidelines) or minimum specifications (self or externally imposed practice rules that are simple and clear). Other local influences, including practitioner-patient interactions and local constraints, can impact self-organized behaviors and produce variability in decision-making.

4. **Decision-Execution Cycles (DEC) **[45]: A decision model developed by Firestone and McElroy that describes how decision cycles help practitioners recognize failures in their current work routines (a single-loop learning opportunity) or the need to reconsider strategies that are guiding routines (a double-loop learning opportunity). In the DEC, practitioners test new information, called knowledge claims (such as new evidence-based innovations), by integrating it into work decisions. When practitioners realize that the expected results of patient care decisions do not match the observed outcomes of those decisions, they form moments of recognition, called epistemic gaps, where single- or double- learning opportunities may occur.

5. **Innovation Diffusion Theory and Dissemination of Innovation **[37,62]: Diffusion theory describes how innovative ideas and practices move (passively) through human systems and the rate of innovation adoption is determined by the characteristics of the adopters. Dissemination strategies are active and explicit attempts by organizations to increase the rate of innovation adoption by its members.

6. **Knowing-doing Gap **[3]: A systemic problem recognized as the failed translation of clinical care research into patient care decisions despite the availability of research knowledge.

7. **Knowledge Management **[8]: Methods that capture and disseminate organizational knowledge to enhance organizational performance.

8. **Learning Organization **[8]: An organization that purposefully designs structures and strategies as to maximize organizational learning.

9. **Loop Learning (single-, double-, and deutero-) **[42]: Organizational learning occurs at three levels of cognitive understanding: 1) learning if work processes are adequate to implement current strategies (single-loop, or "adaptive" learning), 2) learning if current organizational assumptions about strategy effectiveness, patients' need for the strategy, or if the strategy itself should be altered (double-loop, or "generative" learning), and 3) studying the effectiveness of organizational learning processes (deutero-loop learning). In this framework, organizational members act as inquiring agents for the organization by applying organizational knowledge during decision-making with patients and providing feedback about the results of these decisions.

10. **Organizational Culture **[52]: The shared, basic assumptions learned by a group as it strives for external adaptation, internal integration and socialization of new members. Schein identified the overall organizational culture as a source of resistance or acceptance for learning innovative practices, and subculture beliefs within organizations may cause uneven uptake of these innovative practices.

11. **Organizational Knowledge **[8]: The embodied structures of the organization containing the collective understanding, including routines, systems, culture and strategies.

12. **Organizational Knowledge Creation (OKC) **[46]: Members begin the OKC process by applying and recognizing the results of practical applications of knowledge, including evidence-based innovations, during their daily work routines (developing tacit knowledge). Tacit knowledge then must then be negotiated with other team members in order to validate and clarify it prior to disseminating it to the organization (i.e., tacit knowledge becomes explicit and validated).

13. **Organizational Learning **[8]: Knowledge acquisition, dissemination, refinement, creation, and implementation within an organization; the ability to share common understanding so this knowledge can be exploited for the organizations' benefit.

14. **Total Quality Management TQM) **[24]: Described by Deming as a process to improve manufacturing quality and efficiency, TQM is a learning method used by healthcare organizations to identify process changes that will produce an expected improvement in a measurable outcome (e.g., benchmark). TQM views organizations as mechanical systems and uses linear logic models to guide process changes. TQM is useful when certainty of outcomes from decisions is high, processes are mostly linear, and the expected variability in decision-making is low.

## Appendix 2 – Eight screening questions for organizational learning competencies applied to an exemplary patient care team

### Providers

• Are providers asking questions about current practices and finding, appraising and using external knowledge to inform their practices?

◦ **YES: **The exemplary unit is composed of providers (nurses and doctors) who have taken their own time to bookmark evidence-based resources and updates about team-generated best practices on their workstations. They have a workable EBM knowledge and use their resources during point-of-service decisions.

• Are providers effectively deliberating, taking, and evaluating decisions among a viable set of decision options, decision tactics, and potential outcomes?

◦ **YES: **The providers from this unit rely both on the audit reports and their own experiences to provide feedback about the outcomes of their decisions and demonstrate flexibility to consider other decisions when they are required.

• Are providers participating in collaborative relationships and encouraging open dialogue?

◦ **YES: **The providers are remarkably egalitarian; they all demonstrate openness, experimentation, tolerance, and teamwork.

• Are providers forming sensible conclusions about the reasons for the outcomes of their decisions?

◦ **YES: **The providers often make useful suggestions about changes to the unit's work space, work flow, and best practices that will improve care delivery and outcomes.

### Healthcare Teams

• Are practice teams forming and functioning collaboratively?

◦ **YES: **Teams from different shifts are composed of all care providers and deliberate regularly using open discourse with input from all team members.

• Are practice teams deliberating the reasons for patient care outcomes?

◦ **YES: **During deliberations, the teams discuss and debate the conclusions for their failure to achieve their expected outcomes and ways to improve work space, work flow and application of knowledge to achieve the desired outcomes.

• Are teams explicitly sharing their recommendations about needed practice and policy changes with its members, other teams, and organizational leadership?

◦ **YES: **The team's author and share (integrated into their local information system) these conclusions with teams from other shifts and their local managers.

### Organization

• Are organizational leaders providing necessary resources (time, people, information, etc.) and effective motivational strategies to encourage organizational learning, knowledge creation, and knowledge sharing?

◦ **YES: **Although the broader organization is failing to support learning and knowledge management, the managers from this unit use creative time management techniques to create more time for team deliberations, use deft capital project decisions to improve their information systems, demonstrate a collaborative management style, and use thoughtful incentives to induce adaptive practices.
